# Erosive Oral Lichen Planus Treated With Upadacitinib Amid Systemic Psoriatic Therapy

**DOI:** 10.7759/cureus.98831

**Published:** 2025-12-09

**Authors:** Emily Ames, Maggie S Sanders, David Cotter

**Affiliations:** 1 School of Medicine, Kirk Kerkorian School of Medicine at the University of Nevada, Las Vegas, Las Vegas, USA; 2 Dermatology, Las Vegas Dermatology, Las Vegas, USA

**Keywords:** erosive oral lichen planus, jak-stat pathway, mucocutaneous disease, psoriasis, psoriatic arthritis, upadacitinib

## Abstract

Oral lichen planus (OLP) is a rare immune-mediated mucocutaneous disorder with several subtypes, including erosive OLP, which is particularly challenging to manage. First-line therapies are topical corticosteroids or calcineurin inhibitors, while erosive OLP may require intralesional triamcinolone. Refractory OLP requires systemic immunosuppressants. JAK inhibitors are a new consideration for refractory disease. A 41-year-old female with psoriasis (PsO), psoriatic arthritis (PsA), and biopsy-confirmed OLP presented with worsening oral ulceration. Previous treatments, including prednisone, clobetasol gel, dexamethasone, chlorhexidine rinses, and mycophenolate, provided little relief. As her OLP remained unresponsive to treatment, her psoriasis also proved challenging, necessitating multiple therapeutic adjustments. She was started on apremilast for her PsO and PsA, which was ineffective. She transitioned to certolizumab pegol, which provided partial relief but led to recurrent infections. Bimekizumab was then initiated, clearing her PsO, but leaving her with persistent joint pain. Given her persistent PsA, she was started on upadacitinib 15 mg daily, which was later increased to 30 mg. At this dose, her joint pain improved, and her OLP erosions resolved. Upadacitinib, a JAK1 inhibitor approved for PsA, reduces STAT-dependent inflammatory signaling. Studies show JAK1 and JAK3 overexpression in lichen planus (LP) inflammatory infiltrates, implicating JAK signaling in its pathogenesis. Given the inflammatory pathway overlap between PsA and LP, JAK inhibition may mitigate the chronic inflammatory cascade that drives erosive OLP. Upadacitinib’s efficacy in this case of refractory erosive OLP warrants further clinical studies to establish its therapeutic role.

## Introduction

Oral lichen planus (OLP) is a rare chronic mucocutaneous disorder that affects approximately 1% of the global population, with the lowest reported prevalence (~0.47%) in North America [1,2]. It is more prevalent in females and most often affects people between the ages of 30 and 80 years [3]. Although uncommon, malignant transformation occurs in approximately 1.4% of patients with OLP. Increased risk has been associated with smoking, alcohol use, hepatitis C infection, lingual involvement, and the presence of erythematous lesions [2]. OLP is thought to be a T-cell-mediated inflammatory condition. While the antigenic trigger remains unknown, OLP is thought to arise in genetically predisposed individuals in response to factors such as mechanical or chemical irritation, psychological stress, immune or endocrine system dysregulation, and viral infections [4-6].

The clinical presentation of OLP is variable, ranging from asymptomatic white keratotic lesions to painful erosions and ulcerations found in the erosive form of the disease [5]. Erosive OLP can present as an ulcerated red, inflamed area that can contain a white lacy pattern; it is often associated with pain, soreness, or a burning sensation [7]. Diagnosis usually relies on clinical evaluation, with the hallmark finding being reticular white papules or striae (Wickham’s striae) [8]. A biopsy can confirm the diagnosis and reveal hyperkeratosis without parakeratosis, liquefaction of the basal cell layers, saw-toothed rete ridges, Civatte bodies (apoptotic keratinocytes), and a band-like lymphohistiocytic infiltrate at the dermal-epidermal junction [8]. Erosive OLP is a particularly challenging clinical subtype of the condition, as ulceration can interfere with histological features, reducing diagnostic reliability. As such, erosive OLP can be difficult to distinguish from bullous diseases on hematoxylin and eosin staining; however, immunofluorescence can help distinguish between the conditions [9].

Several conditions can mimic OLP either clinically or histologically. Differential diagnoses to consider are oral lichenoid drug reactions, oral lichenoid contact hypersensitivity, mucous membrane pemphigoid, chronic graft-versus-host disease, lupus erythematosus, lichen planus pemphigoides, chronic ulcerative stomatitis, proliferative verrucous leukoplakia, and oral epithelial dysplasia [10]. The diagnosis of OLP requires the clinician to correlate clinical findings with histopathologic evaluation. Histopathologic criteria include features of interface mucositis with a band-like lymphocytic infiltrate and basal cell degeneration. It is also necessary to exclude epithelial dysplasia, verrucous architecture, and deep perivascular inflammatory infiltrates to make a definitive diagnosis. Given the potential for overlap with other inflammatory or premalignant conditions, continued clinical follow-up and, when appropriate, repeat biopsy or immunofluorescence studies may be necessary [10].

Management of OLP is guided by disease severity. Treatment for OLP is initiated when lesions are atrophic, erosive, or symptomatic [8]. Nonerosive OLP is typically managed with high-potency topical corticosteroids, followed by intralesional corticosteroids if necessary [4,8,9]. For severe, refractory disease, systemic agents including methotrexate, mycophenolate mofetil, minocycline, cyclosporine, trimethoprim-sulfamethoxazole, hydroxychloroquine, and acitretin have been used, though overall efficacy data are limited [9,11,12]. A four-to-six-week oral prednisone taper may also be beneficial for individuals with severe OLP [13]. Other systemic medications such as biologics and JAK inhibitors have recently emerged as possible treatments for refractory OLP [9]. We report the successful treatment of refractory erosive OLP in a patient with concurrent psoriasis (PsO) and psoriatic arthritis (PsA) with the selective JAK inhibitor upadacitinib.

## Case presentation

**Figure 1:**
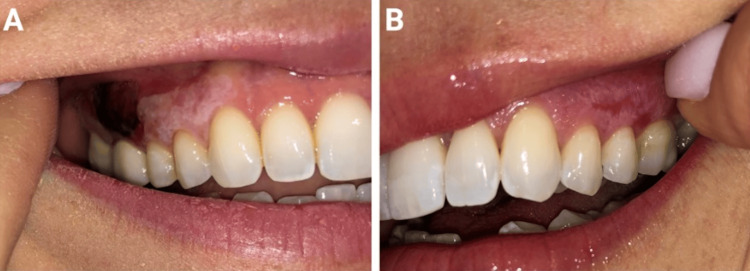
Clinical picture of erosive oral lichen planus before upadacitinib treatment on the patient’s right gingiva (A) and left gingiva (B).

**Figure 2:**
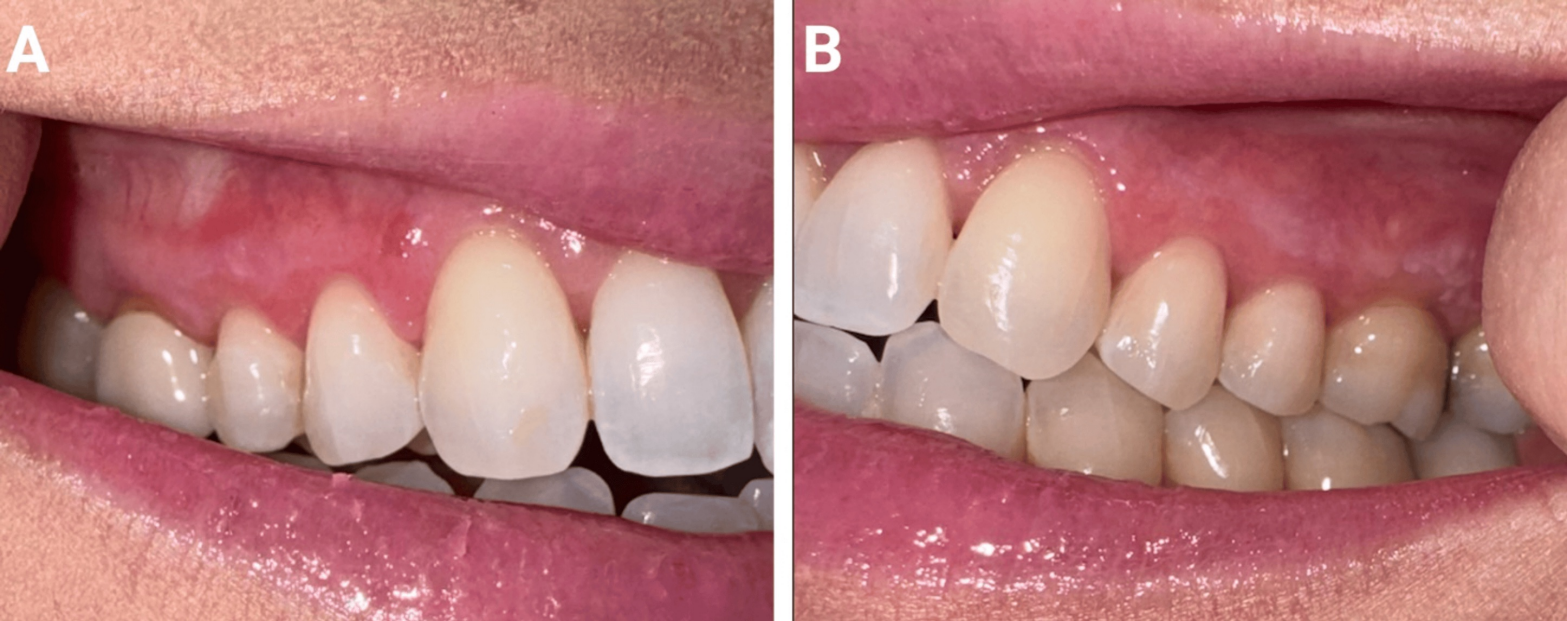
Clinical picture of the patient’s right (A) and left (B) gingiva after treatment with upadacitinib 15 mg daily.

A 41-year-old female with PsO, PsA, and biopsy-confirmed OLP presented with persistent oral ulcerations (Figures 1A, 1B). Her OLP had previously been managed with prednisone and clobetasol 0.05% gel before presentation; however, her symptoms had worsened over the preceding two years. Dexamethasone and chlorhexidine rinses were introduced, but provided little relief. She was started on mycophenolate, but discontinued it after nine months due to minimal improvement. Additionally, her PsO and PsA management also proved challenging, and she failed trials of apremilast, certolizumab pegol, and bimekizumab. Given her persistent PsO and PsA, upadacitinib 15 mg daily was started. At the one-month follow-up, her erosive OLP had resolved, but reticular OLP persisted (Figures 2A, 2B). After six months on upadacitinib 15 mg daily, her joint pain, although improved, persisted, prompting an increase to upadacitinib 30 mg daily, which significantly improved her PsO and PsA symptoms. This adjustment not only alleviated her psoriatic pain but also completely resolved her residual reticular OLP. At her last available follow-up, after a total of 14 months on therapy (eight months on 15 mg and six months on 30 mg daily), her OLP remained asymptomatic, and she experienced no treatment-emergent adverse events. Additionally, missed doses of upadacitinib resulted in recurrence of her OLP within five days, or approximately 10 half-lives, of her last dose. Importantly, reinitiation of upadacitinib resulted in the resolution of her erosive OLP within days without any loss of efficacy.

## Discussion

Erosive OLP is a difficult diagnosis for patients to live with and for clinicians to treat. No single therapy has emerged as the preferred systemic agent for erosive OLP. Varying efficacies and safety and tolerability profiles of medications such as systemic steroids, disease-modifying antirheumatic drugs, and newer options such as apremilast and biologic medications make selecting the appropriate first-line systemic therapy challenging. While the exact pathogenesis of OLP is unclear, current understanding suggests that T lymphocytes activate the JAK pathway, eventually triggering the release of cytokines, including interferon-gamma [14,15]. Studies have shown JAK1 and JAK3 overexpression in OLP lesions, further supporting the role of JAK signaling in its pathogenesis [15]. A recent systematic review supports involvement of JAK-STAT-mediated immune signaling in the pathogenesis of lichen planus, which provides a mechanistic rationale for JAK inhibition in refractory OLP [16]. Recent case reports and small case series have also demonstrated clinical improvement in refractory OLP with the use of JAK inhibitors, further supporting their potential role as a systemic treatment option in severe disease [17].

Upadacitinib, a selective JAK1 inhibitor approved for many T-cell-mediated, inflammatory diseases, reduces STAT-dependent inflammatory signaling and thereby inhibits disease-driving inflammatory cytokines [15,18]. We hypothesize that JAK inhibition, through the use of upadacitinib, mitigated the underlying inflammatory cascade that caused OLP lesions in this patient. In patients with OLP with coexisting PsO and/or PsA, considering systemic immunomodulators such as upadacitinib for treatment of the OLP may provide dual benefit by targeting the common downstream signaling pathways of these conditions.

Limitations of this case report include the small sample size, as it describes a single patient. Additionally, the use of upadacitinib in this case represents an off-label application, and larger studies are needed to further evaluate its safety and efficacy in erosive OLP. This case contributes to the growing body of evidence supporting off-label use of upadacitinib in OLP. In our literature search, we found five previous cases of the successful use of upadacitinib in refractory OLP [19]. Table 1 details these cases.

**Table 1:** Reported cases of upadacitinib use in oral lichen planus. Adapted with modification and permission from Sheehan et al. [19].

Authors	Dose	Outcome summary
Balestri et al. [20]	15 mg once daily	Complete resolution within 7 days
Kooybaran et al. [21]	15 mg once daily	Complete resolution within 24 weeks
Landells et al. [18]	15 mg once daily	Complete resolution (unspecified timeframe), but complicated by oral squamous cell carcinoma
Slater et al. [22]	15 mg once daily	70% resolution within 4 weeks
Sheehan et al. [19]	15 mg once daily	Complete resolution within 4 weeks

As shown in Table 1, Landells et. al reported a case of OLP complicated by oral squamous cell carcinoma [18]. This case displays the clinical importance of the malignant potential of certain subtypes of OLP. Erosive OLP is associated with significant morbidity and reduced quality of life and carries a higher risk of malignant transformation compared with reticular OLP [23].

## Conclusions

This case highlights the rapid and durable response of erosive OLP to upadacitinib. Given the often-refractory nature of OLP and high morbidity, the efficacy of JAK inhibitors such as upadacitinib warrants further clinical research to establish its therapeutic role in disease management. There has been one reported case of recalcitrant erosive OLP successfully treated with upadacitinib that was later complicated by oral squamous cell carcinoma. It is unclear if the malignant transformation was secondary to upadacitinib use or from chronic inflammation; however, additional research is needed to ensure the safety of its use in OLP. Given the off-label use of upadacitinib, regular surveillance is advised, and a low threshold to biopsy persistent lesions, especially given the potential associated risk of malignant transformation.
